# Supplementary material for: The Effect of Acidic and Alkaline Seawater on the F-Actin-Dependent Ca2+ Signals Following Insemination of Immature Starfish Oocytes and Mature Eggs
Source: Cells. 2023 Feb 25;12(5):740. doi: 10.3390/cells12050740 (PMC10000582; doi:10.3390/cells12050740)
Supplement: Supplementary file 1 [file cells-12-00740-s001.zip › Figure S1.pdf]

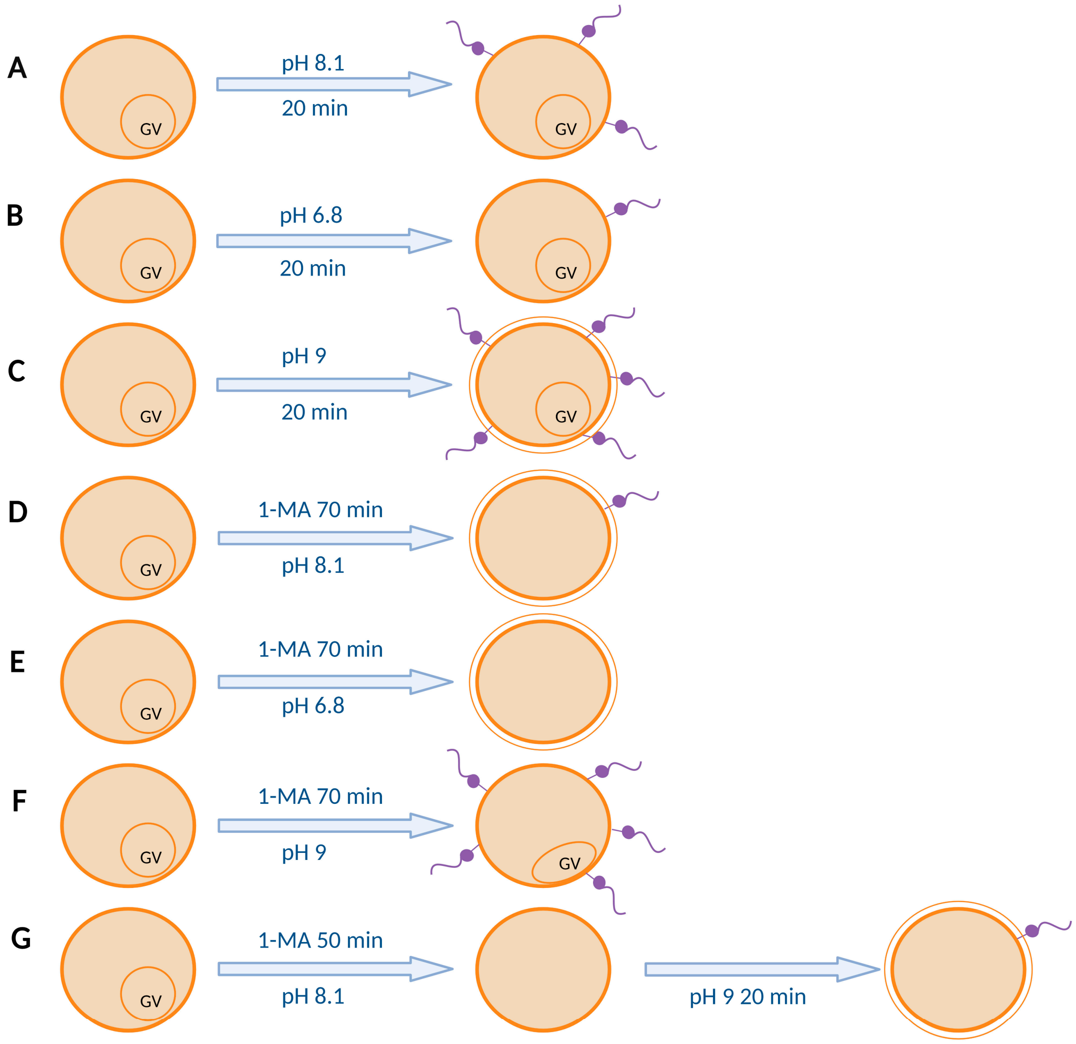

Figure S1. Summary of the experimental design. (A-C) GV-stage oocytes were incubated in seawater at different pH for 20 min and then inseminated in the same medium. (D-F) GV-stage oocytes were first matured with 1-methyladenine (1-MA) in seawater at different pH and subsequently inseminated in the same media. (G) GV-stage oocytes were first stimulated with 1-MA in natural seawater (pH 8.1) for 50 min but exposed to seawater (pH 9.0) for 20 min before the insemination in the alkaline seawater.
