# Supplementary material for: The Effect of Acidic and Alkaline Seawater on the F-Actin-Dependent Ca2+ Signals Following Insemination of Immature Starfish Oocytes and Mature Eggs
Source: Cells. 2023 Feb 25;12(5):740. doi: 10.3390/cells12050740 (PMC10000582; doi:10.3390/cells12050740)

Reagents and solvents were purchased from Sigma-Aldrich, TCI, Fluorochem and were used without further purification. Deuterated solvents were purchased from Euriso-TOP or Sigma-Aldrich.

Flash column chromatography was performed a Biotage Isolera with a SNAP Ultra C18 column.

NMR (ISIS, Univ. of Strasbourg): Nuclear magnetic resonance (NMR) data were collected on Bruker AVANCE III 400 (400 MHz) Spectrometer equipped with a Prodigy BBO probe.

LC-HRMS (ESI+) (ISIS, Univ. of Strasbourg): Analyses were performed using a Dionex RSLC U3000HPLC system (Thermo) with a chromatography column Thermo Fisher HyperGOLDSIL C18 (50x2.1mm, 1.9 µm). The mobile phase was water with 0.1% formic acid (method A) or acetonitrile with 0.1% formic acid (method B). Full MS spectra were acquired using Exactive series 2.9 sp4 software in a positive ion mode at a 3.5 kV spray voltage setting on a Thermo Scientific Exactive Plus EMR. Resolution of full MS and HCD scans were 140,000 and data were acquired in profile mode and processed using Xcalibur 4.3.

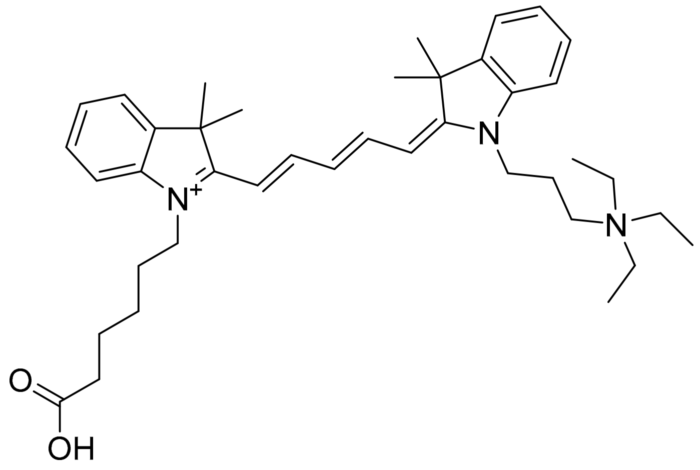

1-(5-carboxypentyl)-2-((1E,3E)-5-((E)-3,3-dimethyl-1-(3-(triethyl-l4-azaneyl)propyl)indolin-2-ylidene)penta-1,3-dien-1-yl)-3,3-dimethyl-3H-indol-1-ium.

To a round bottom flask were added 1-(5-carboxypentyl)-2,3,3-trimethyl-3H-indol-1-ium bromide (1 eq., 123 mg, 0.45 mmol) , N-[(1E,3E)-3-(phenylimino)prop-1-en-1-yl]aniline (1 eq., 100 mg, 0.45 mmol), acetic acid (4.75 mL) , and acetic anhydride (11.2 eq., 516 mg, 0.475 mL, 5.05 mmol). The resulting dark red solution was heated up to 120°C for 4 hours. Then 2,3,3-trimethyl-1-[3-(triethylazaniumyl)propyl]-3H-indol-1-ium (1.1 eq., 149 mg, 0.495 mmol) was added to this solution in acetic acid (2 mL) followed by potassium acetate (8.71 eq., 384 mg, 3.92 mmol) resulting in an immediate colour change to dark blue. The reaction mixture was heated to 120°C and stirred overnight. Evaporation of the solvent under reduced pressure at 60°C, followed by flash chromatography purification, dry loading prepared from a methanol solution of the crude using celite, column used: SNAP Ultra C18 (reversed phase) 60g, gradient water (10% acetic acid) --> water (10% acetic acid)/MeOH. Then dried under high vacuum (115.5 mg, 0.19 mmol, 42%).

^{1}H NMR (400 MHz, MeOD) δ 8.30 (dt, *J* = 26.4, 13.0 Hz, 2H), 7.60 – 7.21 (m, 8H), 6.71 (t, *J* = 12.5 Hz, 1H), 6.45 (d, *J* = 13.9 Hz, 1H), 6.28 (d, *J* = 13.3 Hz, 1H), 4.59 (s, 6H), 4.19 (q, *J* = 8.4 Hz, 4H), 3.46 – 3.34 (m, 6H), 2.26 – 2.15 (m, 3H), 1.95 (s, 6H), 1.87 (t, *J* = 7.6 Hz, 1H), 1.76 (s, 6H), 1.72 (d, *J* = 5.2 Hz, 1H), 1.56 – 1.48 (m, 1H), 1.33 (t, *J* = 7.3 Hz, 9H).

HRMS (ESI+): [M+H$^+$] calc. for [$C_{40}H_{57}N_3O_2$]: 610.43780 Da; found: 610.4358

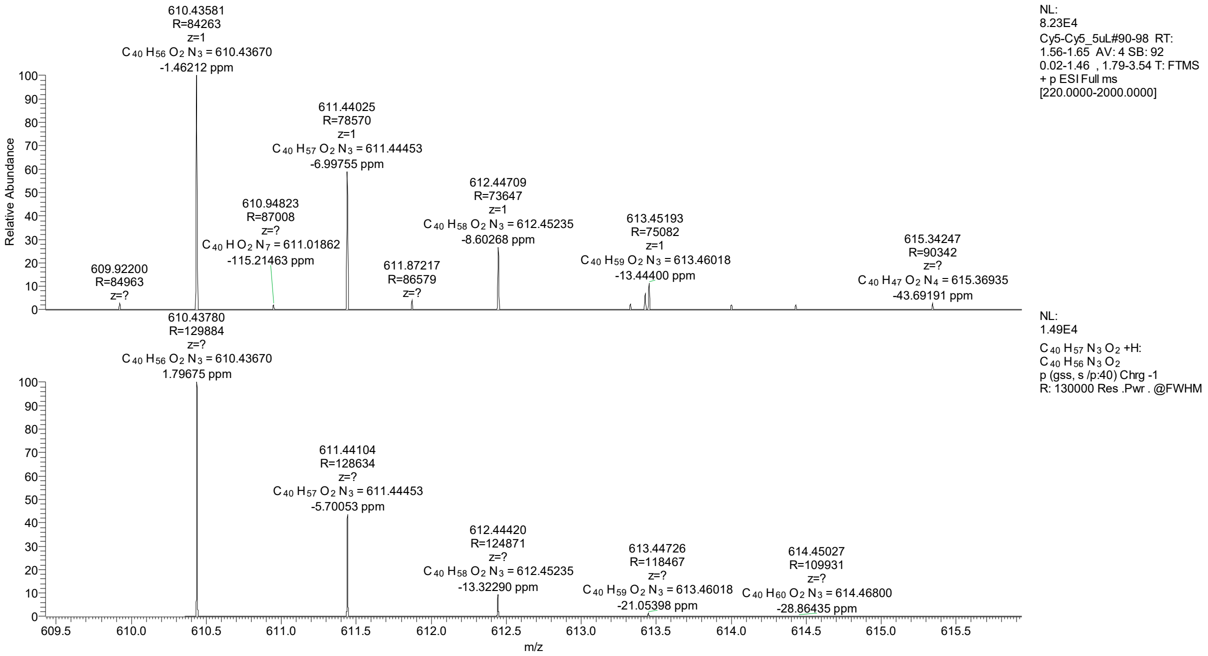

HRMS (ESI+): [M+H$^+$] calc. for [$C_{40}H_{57}N_3O_2$]: 305.7219 Da; found: 305.7218

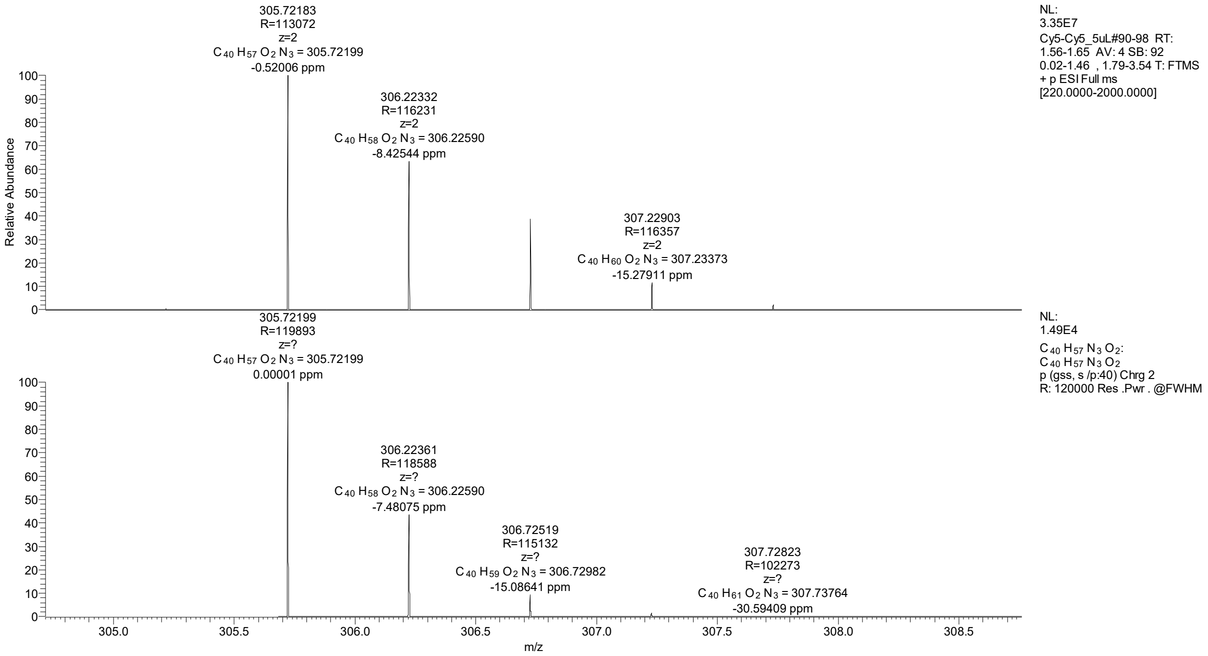

Cy5 UV trace from the HPLC Diode array detector

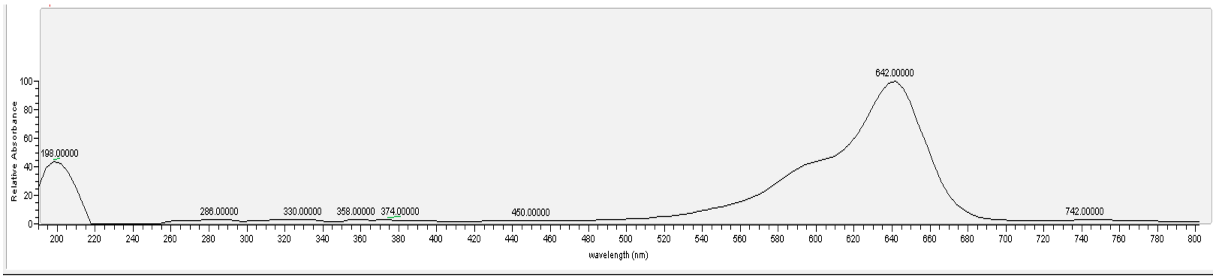

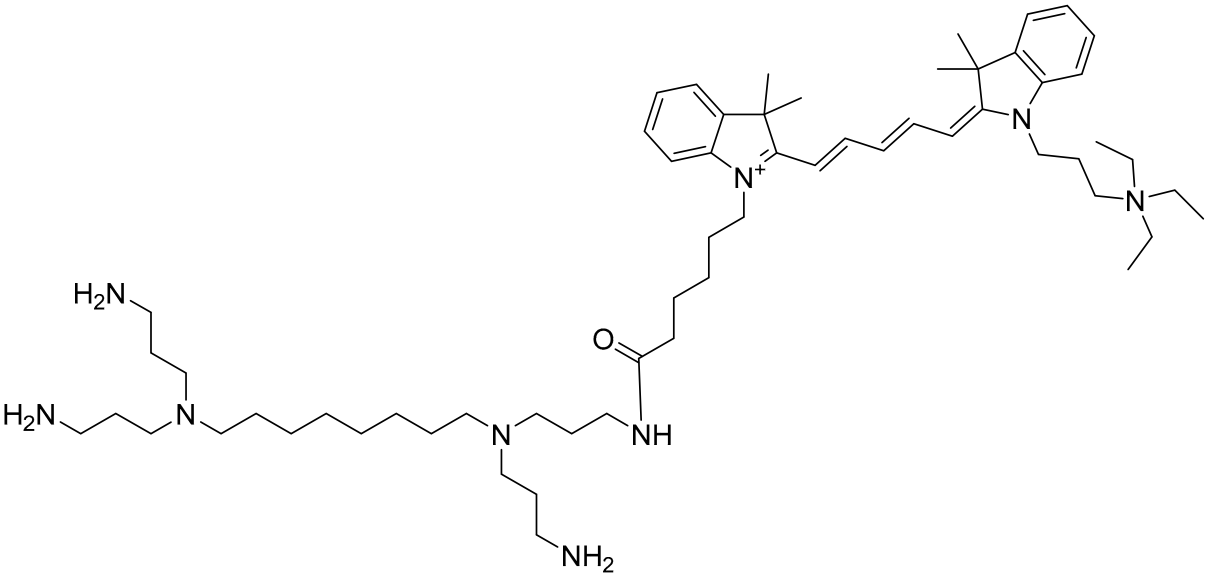

1-(6-((3-((3-aminopropyl)(8-(bis(3-aminopropyl)amino)octyl)amino)propyl)amino)-6-oxohexyl)-2-((1E,3E)-5-((E)-3,3-dimethyl-1-(3-(triethyl-l4-azaneyl)propyl)indolin-2-ylidene)penta-1,3-dien-1-yl)-3,3-dimethyl-3H-indol-1-ium

Solution A:

To a solution of Cy-5 (2-[(1E,3E)-5-[(2E)-1-(5-carboxypentyl)-3,3-dimethyl-2,3-dihydro-1H-indol-2-ylidene]penta-1,3-dien-1-yl]-3,3-dimethyl-1-[3-(triethylazaniumyl)propyl]-3 (1 eq., 176 mg, 0.289 mmol)) and TSTU (TSTU (2 eq., 173 mg, 0.577 mmol)) in DMF (0.7 ml) was added triethylamine (2 eq., 58.4 mg, 80.3 µL, 0.577 mmol) and the solution was stirred at rt during 2h. (no color changing). Rxn followed by LC/MS.

Solution B:

A solution of bis(3-aminopropyl)({8-[bis(3-aminopropyl)amino]octyl})amine (2 eq., 215 mg, 0.577 mmol) (hexa HCl salt, actual weight measured: 341.8 mg and Na2CO3 (10 eq., 305 mg, 2.89 mmol) in water (1.1 mL) was prepared.

After 2h, **Solution A** was added to **Solution B** using water (195 µL) under stirring. The final mixture was stirred at rt for 15h.Evaporation of the solvent under reduced pressure at 60°C,

followed by flash chromatography purification, dry loading prepared from a methanol solution of the crude using celite, column used: SNAP Ultra C18 (reversed phase) 60g, gradient water (10% acetic acid) --> water (10% acetic acid)/MeOH. Then dried under high vacuum (4.5 mg, 0.046 mmol, 9%).

HRMS (ESI+): [M^+] calc. for [$C_{60}H_{103}N_9O$]: 482.9137 Da; found: 482.9137

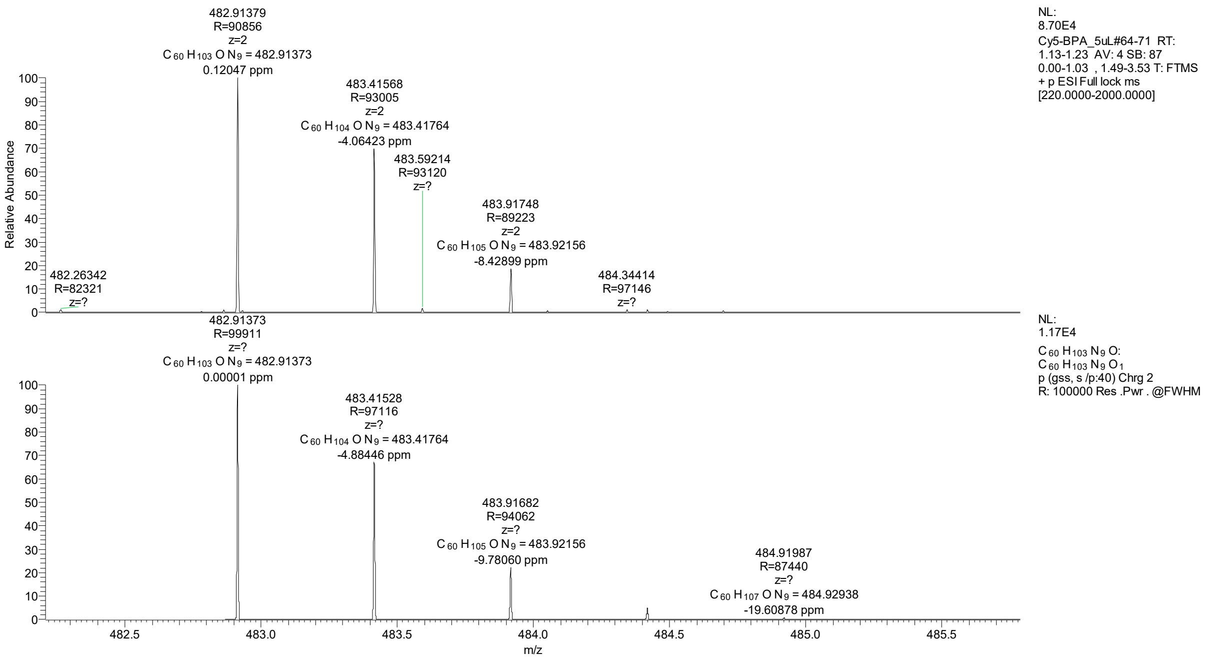

HRMS (ESI+): [$M+H^+$] calc. for [$C_{60}H_{103}N_9O+H^+$]: 322.2782 Da; found: 322.2781

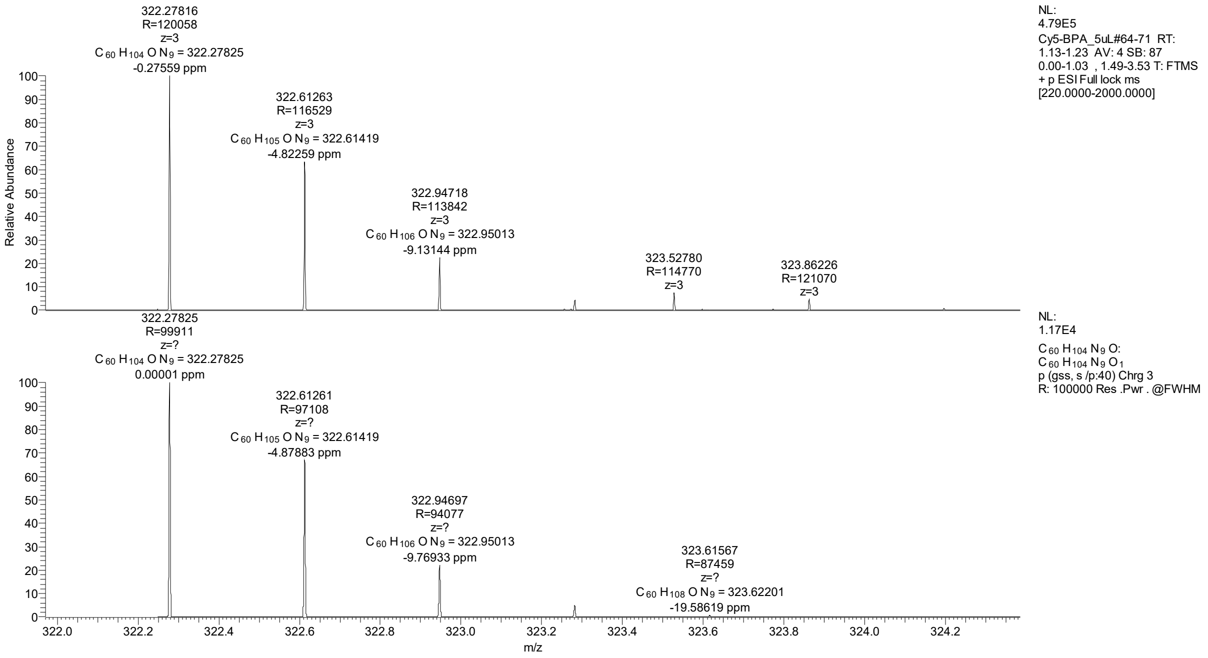

BPA-Cy5 UV trace from the HPLC Diode array detector

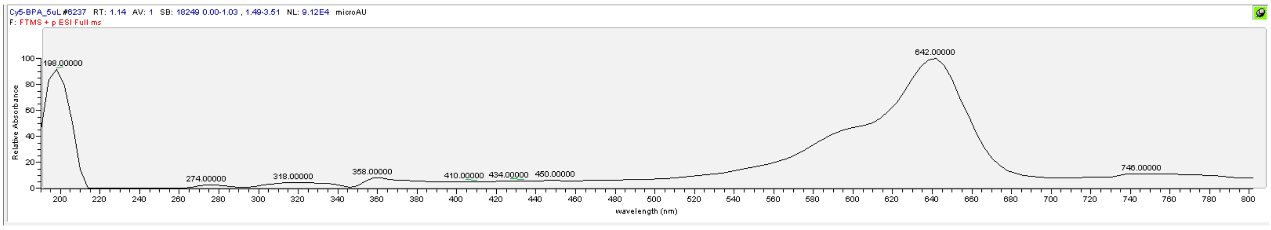

Supplement: Supplementary file 1 [file cells-12-00740-s001.zip › Supplementary File 1.pdf]
